# Craniofacial characteristics of Syrian adolescents with Class II division 1 malocclusion: a retrospective study

**DOI:** 10.7717/peerj.9545

**Published:** 2020-07-15

**Authors:** Alaa Al Ayoubi, Daniel Dalla Torre, Melinda Madléna

**Affiliations:** 1 Department of Orthodontics and Pediatric Dentistry, Faculty of Dentistry, University of Szeged, Szeged, Hungary; 2 University Clinic of Craniomaxillofacial Surgery, Medical University Innsbruck, Innsbruck, Austria

**Keywords:** Dentofacial morphology, Upper airway, Tooth size, Class II division 1 malocclusion, Syrian adolescents

## Abstract

**Background:**

Malocclusion characteristics vary across different ethnic groups and populations. Limited data are available regarding the characteristics of Syrian adolescents with Class II division 1 (Class II-1) malocclusion, and the recent inflow of Syrian refugees and immigrants into Europe and many areas worldwide demonstrate the need for updated studies to discover the craniofacial characteristics of these new immigrants.

**Objectives:**

The present compound cephalometric and tooth-size study sought to assess the dentofacial morphology, upper-airway dimensions, and tooth-size characteristics of Syrian adolescents with Class II-1 malocclusion and compare the results with established Syrian population norms.

**Materials and Methods:**

The study sample consisted of 43 Syrian patients including 24 females and 19 males with Class II-1 malocclusion (age: 14.3 (±1.5) years, mean (±SD)). Cephalometric radiographs and orthodontic casts were analyzed using special orthodontic software (OnyxCeph3^™^) and a universal digital caliper, respectively. Statistics were calculated using the SPSS software.

**Results:**

In Syrian adolescents with Class II-1 malocclusion, the position of the mandible relative to the nasion perpendicular (mean (95% confidence interval)) was −11.01 (−12.45, −9.57) mm. Facial axis angle showed a negative value: −6.25 (−7.65, −4.85) degrees. An obtuse nasolabial angle was observed: 104.05 (101.77, 106.33) degrees. The average width of the upper pharynx was 11.50 (10.53, 12.47) mm; however, there was no prevalence of an upper-pharyngeal width of 5 mm or less. The average value of the anterior tooth-size ratio was 80.69 (79.85, 81.53) percent. In total, 39.5% of the investigated subjects had anterior ratios outside two standard deviations from Bolton’s norm, while 25.6% of the investigated subjects had anterior ratios outside two standard deviations from Syrian population norm.

**Conclusions:**

In this study, the inter-maxillary discrepancy of Class II-1 Syrian adolescents was a consequence of their hyperdivergent facial pattern. The observed small pharyngeal widths were not clinically significant, while the anterior tooth-size discrepancy might be clinically relevant.

## Introduction

In orthodontics, it is essential to understand the complex relationship between skeletal, dental and facial aberrations in each malocclusion to achieve an accurate diagnosis followed by an optimal treatment plan. Class II division 1 (Class II-1) malocclusion has been suggested as the most frequent pathology that orthodontists may encounter in their practice. According to epidemiologic studies conducted among different populations, the prevalence of this malocclusion ranges from 12 to 40% worldwide (Table S1) (Massler & Frankel, 1951; Thilander & Myrberg, 1973; Foster & Walpole Day, 1974; El-Mangoury & Mostafa, 1990; Lew, Foong & Loh, 1993; Saleh, 1999; Silva & Kang, 2001; Thilander et al., 2001; Onyeaso, 2004; Tausche, Luck & Harzer, 2004; Gábris, Márton & Madléna, 2006; Borzabadi-Farahani, Borzabadi-Farahani & Eslamipour, 2009; Perillo et al., 2009; Bugaighis & Karanth, 2013; Alatrach, Saleh & Osman, 2014; Nadim, Aslam & Rizwan, 2014; Singh & Sharma, 2014; Bilgic, Gelgor & Celebi, 2015; De Souza et al., 2016; Albakri, Ingle & Assery, 2018; Shyagali et al., 2019).

Numerous studies have investigated the cephalometric and tooth-size characteristics of patients with Class II-1 malocclusion using various measurements either on cephalometric radiographs or orthodontic casts. However, the significance of these studies is limited by multiple factors, including the conflicting results; some cephalometric studies have reported that a retrognathic mandible is the key factor contributing to Class II-1 malocclusion with a normally positioned maxilla (Riedel, 1952; Hitchcock, 1973; Freitas et al., 2005; Sayın & Türkkahraman, 2005; Isik et al., 2006). In contrast, other studies have indicated that maxillary protrusion is the most common feature of Class II-1 malocclusion with a neutral mandibular position (Altemus, 1955; Rothstein & Yoon-Tarlie, 2000). Finally, some researchers have described the malpositioning of both the maxilla and mandible in this condition (Drelich, 1948; Lau & Hägg, 1999; Al-Khateeb & Al-Khateeb, 2009). Likewise, inconsistent results have been reported regarding the relationship between Class II-1 malocclusion and upper-airway dimensions, where some investigators suggested Class II-1 malocclusion may have an influence on the upper-airway dimensions (Mergen & Jacobs, 1970; Silva et al., 2015; Soni et al., 2015) while other authors did not find a significant correlation between the two (Sosa, Graber & Muller, 1982; Ceylan & Oktay, 1995; Bollhalder et al., 2013). Further, a number of tooth-size studies have investigated Class II-1 malocclusion; their results were also contradictory, with some reporting significant differences in tooth-size ratios between Class II-1 malocclusion and Class I malocclusion or normal occlusion (Fattahi, Pakshir & Hedayati, 2006; Wędrychowska-Szulc, Janiszewska-Olszowska & Stepień, 2010; Mollabashi et al., 2019) and others indicating no significant differences in this regard (Crosby & Alexander, 1989; Oktay & Ulukaya, 2010; Machado et al., 2018).

It is also important to consider that most of these previous studies were performed on radiographs or casts of Caucasians or without mention of the ethnicity of the investigated subjects, ignoring the fact that ethnicity is an important etiologic factor in Class II malocclusion (Lau & Hägg, 1999; Ishii, Deguchi & Hunt, 2002). Because of recent wars and conflicts, such as the Syrian conflict, ethnic diversity is increasing worldwide. During the last few years, Syrian refugees and immigrants have occupied the foreground of migration statistics, particularly in Europe (Eurostat Database, 2019). Data show that the population of Syrian immigrants has reached 8.2 million in 2019 and is considered to be among the fastest-growing populations of ethnic minorities (DESTATIS, 2018; United Nations Population Division, 2019). This inflow of Syrian refugees and immigrants into Europe and in many areas worldwide demonstrate the need for updated studies to discover the craniofacial characteristics of these new immigrants. Former studies on the Syrian population aimed to establish cephalometric and tooth-size norms (Nourallah et al., 2005; Al Sabbagh, 2014). However, limited data are available on the characteristics of Syrian adolescents with Class II-1 malocclusion.

The objective of this study was to elucidate the cephalometric and tooth-size characteristics of Syrian adolescents with Class II-1 malocclusion and compare the acquired data with those of Syrian population norms.

## Materials and Methods

### Subjects

Ethical approval for the present retrospective study was obtained from the Human Investigation Review Board at the University of Szeged (151/2018-SZTE). Written informed consent to participate in this study was provided by the participants’ guardians/parents.

According to previous dentofacial, upper-airway, and tooth-size studies, effect sizes were estimated from the Pog-Np distance 7.8 (±8.1) mm (Sayın & Türkkahraman, 2005), the upper-pharyngeal width 3.1 (±2.6) mm (Mergen & Jacobs, 1970), and the upper first molar’s width 0.2 (±0.2) mm (Lavelle, 1972). On the basis of a significance level of alpha of 0.05 (two-sided) with a power of 80%, the sample size was calculated to detect the standardized effect sizes of 0.96 (7.8/8.1 mm), 1.19 (3.1/2.6 mm) and 1 (0.2/0.2 mm) for dentofacial, upper-airway and tooth-size comparisons, respectively. Sample size calculations when considering these three types of comparisons showed that 17 patients of each gender were necessary for inclusion in the present study (Hulley et al., 2013). As a consequence, cephalometric radiographs and orthodontic casts of 43 untreated patients with skeletal and dental Class II-1 malocclusion (24 females and 19 males, age: 14.3 (±1.5) years, mean (±standard deviation)) were selected from a private orthodontic practice in Damascus, Syria, based on the following inclusion criteria:

- Adolescents of Syrian origin aged between 12 and 17 years
- Overjet of more than 4 mm with an absence of retroclination of the upper incisors (1U/NA angle ≥ 22 degrees)
- Permanent dentition with bilateral distal occlusion (half-unit or greater)
- ANB angle of more than 4 degrees with a convex facial profile
- Absence of extractions or interproximal caries/restorations or any other condition that affects the dental mesiodistal distance

Patients with craniofacial syndromes or a history of trauma as well as previous orthodontic treatment were excluded from this study. Additionally, we excluded patients with cephalograms in which a swallowing action or obvious hyperplasia of tonsils and adenoids was detected.

Two previous studies on the Syrian population were used as sources of normative cephalometric and tooth-size measurements (Nourallah et al., 2005; Al Sabbagh, 2014) (Table S2).

Age and gender distributions in the present study sample and normative studies’ samples used as sources of normative measurements are shown in Table S3.

### Cephalometric measurements

**Figure 1:**
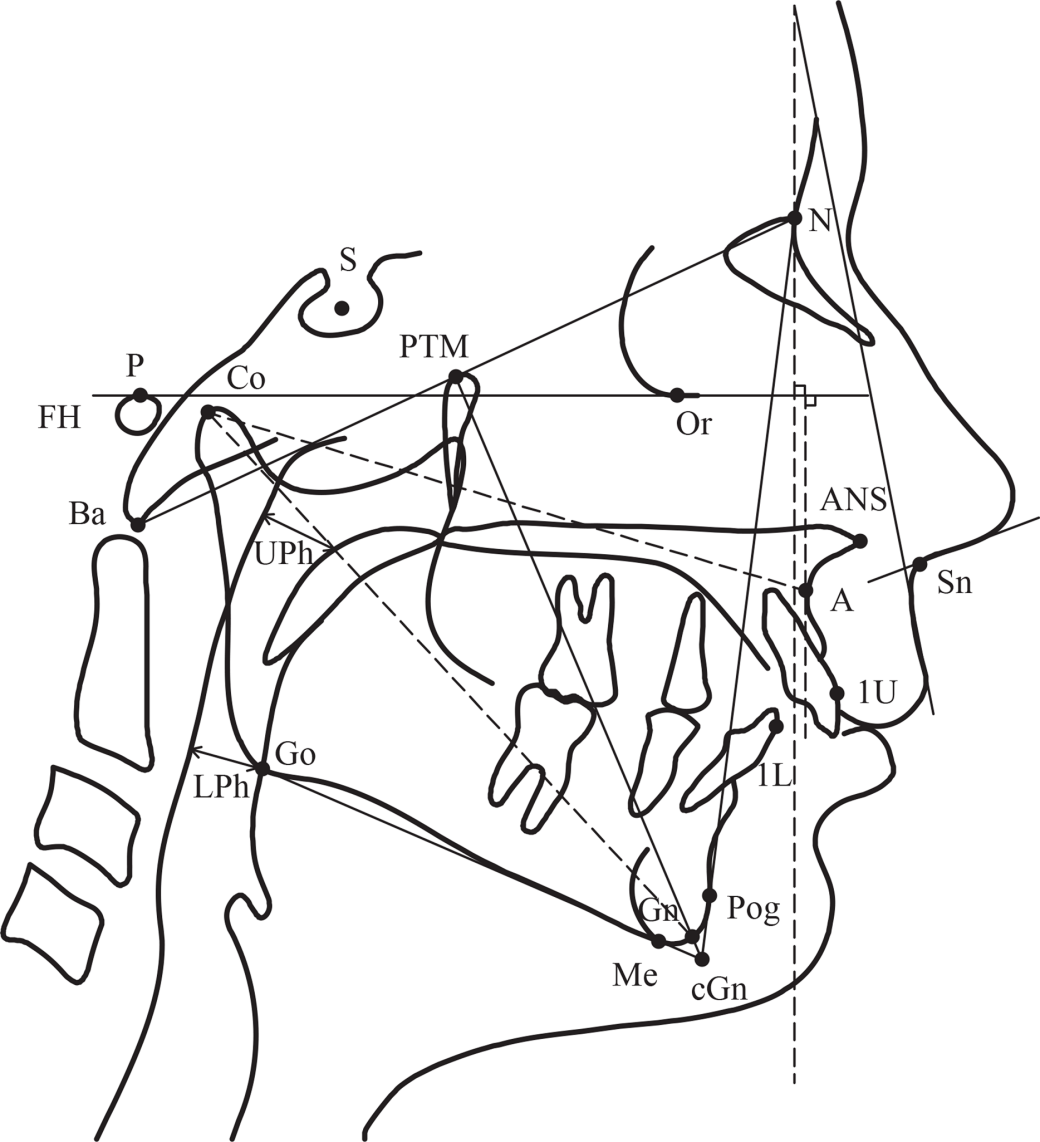
Reference cephalometric landmarks and lines used in this study.

Pretreatment lateral cephalometric radiographs were taken for each patient with the head in the natural position using a dental radiograph system (PAX 400; Vatech Co., Hawseong, Korea). The same *X*-ray machine was used to acquire cephalograms in the normative cephalometric study using the same protocol (Al Sabbagh, 2014). The cephalometric measurements used in this study were derived from the analyses developed by McNamara (1984) and McNamara, Brust & Riolo (1992). Reference landmarks and lines are illustrated in Fig. 1. Definitions of the cephalometric measurements are shown in Table S4. Each radiograph was digitized and analyzed by one investigator (A. A.) using a special orthodontic software program (OnyxCeph3^™^, Image Instruments GmbH, Chemnitz, Germany). Additionally, upper-pharyngeal widths were computed for all Class II-1 subjects whose values were equal to 5 mm or less (McNamara, 1984).

### Orthodontic cast measurements

The mesiodistal crown diameters of all teeth from the right first permanent molar to the left first permanent molar on each cast were measured to the nearest 0.01 mm by one investigator (A. A.) using a universal digital caliper (MIB Messzeuge GMBH, Spangenberg, Germany). The measurements were conducted according to the methods described by Seipel (1946) and Moorrees & Reed (1964). The same method was used in the normative tooth-size study (Nourallah et al., 2005). Bolton’s overall ratio (sum of mesiodistal widths of 12 mandibular teeth divided by the sum of mesiodistal widths of 12 maxillary teeth multiplied by 100) and Bolton’s anterior ratio (sum of mesiodistal widths of six mandibular anterior teeth divided by the sum of mesiodistal widths of six maxillary anterior teeth multiplied by 100) were calculated and used in the statistical analysis (Bolton, 1958). Overall and anterior ratios were computed for all Class II-1 subjects whose values were outside two SDs from Bolton’s norms (Bolton, 1958) and were also computed for all Class II-1 subjects whose values were outside two SDs from Syrian population norms (Nourallah et al., 2005).

### Method error

Following a random selection of 10 patients, all measurements on their cephalometric radiographs and orthodontic casts were retaken 2 months later by the same investigator. To evaluate the method error, both measurements were compared using three approaches.

First, the random method error was established according to Dahlberg’s formula (Dahlberg, 1940). The method errors for angular and linear measurements on cephalometric radiographs were within 0.64 degrees and 0.42 mm, respectively, while the method error for tooth-size measurements on orthodontic casts did not exceed 0.19 mm, and the errors of anterior and overall tooth-size ratios were 0.62 and 0.39, respectively. Second, the systematic error was investigated by paired sample *t-tests;* results showed that no systematic error could be found (*p* > 0.05). Finally, intraclass correlation coefficients were calculated for all variables; results ranged from 0.935 to 0.999.

Inter-examiner reliability was established to investigate the potential investigator bias. Measurements of 10 randomly selected cephalograms and casts were replicated for a second time by another investigator. Random errors were within 0.53 mm and 0.61 degrees for linear and angular cephalometric variables, respectively, and within 0.28 mm for tooth-size measurements. Random errors of anterior and overall tooth-size ratios were 0.56 and 0.47, respectively. Systematic error was absent (*p* > 0.05) and intraclass correlation coefficients were above 0.92.

### Statistical analysis

Descriptive statistics (Means, SDs and 95% CIs) of all variables were calculated with the use of the SPSS software 24.0 (SPSS Inc., Chicago, IL, USA). All variables in the total group (43 Syrian adolescents with Class II-1 malocclusion) and within each gender group (24 Syrian females and 19 Syrian males) were normally distributed according to Shapiro–Wilk test.

## Results

Data presenting gender-based and general characteristics of Syrian adolescents with Class II-1 malocclusion are summarized in Table 1.

**Table 1:** Cephalometric measurements and tooth-size ratios of Syrian adolescents with Class II-1 malocclusion.

Cephalometric measurements	Syrian Males with Class II-1 Malocclusion (*n* = 19)		Syrian Females with Class II-1 Malocclusion (*n* = 24)		Syrian adolescents with Class II-1 Malocclusion (*n* = 43)	
Variables	Mean (±S.D)	95% CIs for mean Lower, Upper	Mean (±S.D)	95% CIs for mean Lower, Upper	Mean (±S.D)	95% CIs for mean Lower, Upper
Skeletal measurements						
Sagittal values						
A-NP (mm)	−0.64 (±2.60)	**−1.90, 0.61**	0.53 (±2.00)	**−0.31, 1.38**	0.01 (±2.33)	**−0.70, 0.73**
SNA (°)	79.70 (±2.72)	**78.39, 81.01**	81.06 (±2.50)	**80.00, 82.11**	80.46 (±2.66)	**79.64, 81.27**
Pog-NP (mm)	−10.95 (±3.85)	**−12.81, −9.10**	−11.05 (±5.34)	**−13.31, −8.80**	−11.01 (±4.69)	**−12.45, −9.57**
Cond-A (mm)	85.84 (±5.31)	**83.28, 88.39**	85.25 (±3.79)	**83.65, 86.85**	85.51 (±4.48)	**84.13, 86.89**
Cond-Gn (mm)	108.47 (±5.81)	**105.67, 111.27**	107.59 (±5.43)	**105.30, 109.89**	107.98 (±5.55)	**106.27, 109.69**
Max-Mand (mm)	22.64 (±4.26)	**20.59, 24.69**	22.34 (±4.59)	**20.41, 24.28**	22.47 (±4.39)	**21.12, 23.82**
Vertical values						
ANS-Me (mm)	68.65 (±5.41)	**66.04, 71.26**	68.01 (±4.92)	**65.94, 70.09**	68.30 (±5.09)	**66.73, 69.86**
MP-FH (°)	28.70 (±5.15)	**26.21, 31.18**	30.61 (±5.94)	**28.11, 33.12**	29.77 (±5.62)	**28.04, 31.50**
Facial Axis (°)	−6.35 (±5.05)	**−8.78, −3.91**	−6.18 (±4.24)	**−7.97, −4.38**	−6.25 (±4.56)	**−7.65, −4.85**
Dental measurements						
1U-AP (mm)	5.66 (±2.07)	**4.66, 6.65**	6.03 (±2.06)	**5.16, 6.90**	5.86 (±2.05)	**5.23, 6.49**
1L-APog (mm)	4.33 (±1.92)	**3.40, 5.25**	5.27 (±1.79)	**4.51, 6.02**	4.85 (±1.89)	**4.27, 5.43**
Soft tissue measurements						
NLA (°)	104.78 (±6.32)	**101.73, 107.82**	103.47 (±8.27)	**99.98, 106.96**	104.05 (±7.42)	**101.77, 106.33**
UL-NP (°)	9.16 (±7.51)	**5.54, 12.78**	12.54 (±7.49)	**9.38, 15.70**	11.05 (±7.60)	**8.71, 13.39**
Airway measurements						
UPh (mm)	10.57 (±3.17)	**9.04, 12.10**	12.24 (±3.00)	**10.97, 13.51**	11.50 (±3.15)	**10.53, 12.47**
LPh (mm)	10.44 (±2.48)	**9.25, 11.63**	11.41 (±3.19)	**10.06, 12.75**	10.98 (±2.90)	**10.09, 11.87**

Tooth-size ratios	Syrian Males with Class II-1 Malocclusion (*n* = 19)		Syrian Females with Class II-1 Malocclusion (*n* = 24)		Syrian adolescents with Class II-1 Malocclusion (*n* = 43)	
Variables	Mean (±S.D)	95% CIs for means Lower, Upper	Mean (±S.D)	95% CIs for means Lower, Upper	Mean (±S.D)	95% CIs for means Lower, Upper
Anterior ratio (%)	80.55 (±2.95)	**79.12, 81.97**	80.81 (±2.60)	**79.71, 81.91**	80.69 (±2.73)	**79.85, 81.53**
Overall ratio (%)	92.74 (±1.80)	**91.87, 93.61**	92.92 (±1.65)	**92.23, 93.62**	92.84 (±1.70)	**92.32, 93.37**

**Notes:**

CIs, confidence intervals; S.D, standard deviation.

Bold values indicate lower and upper bounds of 95% confidence intervals for mean.

Two variables (A-NP, SNA) were used to assess the sagittal position of the maxilla. The linear variable (A-NP) showed a value of 0.01 (±2.33) mm. The angular variable (SNA) showed a value of 80.46 (±2.66) degrees. The sagittal mandibular position was determined by one linear variable (Pog-NP) with a value of −11.01 (±4.69) mm. The effective length of the maxilla (Cond-A) was 85.51 (±4.48) mm. The effective length of the mandible (Cond-Gn) was 107.98 (±5.55) mm. The difference between the maxillary length and the mandibular length (Max-Mand) was 22.47 (±4.39) mm.

Results in the vertical plane were as follows: First, lower anterior facial height (ANS-Me) showed a value of 68.30 (±5.09) mm. Second, mandibular plane angle (MP-FH) showed a value of 29.77 (±5.62) degrees. Third, facial axis (facial axis) showed a value of −6.25 (±4.56) degrees.

For incisors position determination, two linear variables were used: First, maxillary incisors position (1U-AP) showed a value of 5.86 (±2.05) mm. Second, mandibular incisors position (1L-APog) showed a value of 4.85 (±1.89) mm.

Soft tissue measurements showed an obtuse nasolabial angle (NLA) of 104.05 (±7.42) degrees. Further, the value of the upper-lip angle (UL-NP) was 11.05 (±7.60) degrees.

Regarding upper-airway dimensions, upper and lower-pharyngeal widths (UPh, LPh) were assessed. The upper-pharyngeal width (UPh) was 11.50 (±3.15) mm. The lower-pharyngeal width (LPh) was 10.98 (±2.90) mm. However, there was no prevalence of upper-pharyngeal obstructions (upper-pharyngeal width ≤ 5 mm) in the subjects of this study.

Bolton tooth-size analysis revealed that the anterior ratio was 80.69 (±2.73) percent, while the overall ratio was 92.84 (±1.70) percent.

The percentage of Class II-1 patients who had anterior ratios greater than two SDs from Bolton’s norm (77.2 (±1.65) percent) (Bolton, 1958) was 39.5%, whereas the percentage of Class II-1 patients who had anterior ratios greater than two SDs from Syrian population norm (78.99 (±2.18) percent) (Nourallah et al., 2005) was 25.6%. The percentage of Class II-1 patients who had overall ratios greater than two SDs from Bolton’s norm (91.3 (±1.91) percent) (Bolton, 1958) was 6.98%, whereas none of Class II-1 patients had overall ratios greater than two SDs from Syrian population norm (92.26 (±2.06) percent) (Nourallah et al., 2005). Additionally, none of Class II-1 patients had anterior or overall ratios smaller than two SDs from Bolton’s norms (Bolton, 1958), and none of Class II-1 patients had anterior or overall ratios smaller than two SDs from Syrian population norms (Nourallah et al., 2005).

## Discussion

Several studies have reported the cephalometric and tooth-size features of individuals of Middle Eastern ethnicity (Al Jundi & Riba, 2014; Ali, El-Shorbagy & Elliathy, 2016; Fouda, Hafez & Al-Awdi, 2017; Mollabashi et al., 2019; ElAbbasy, 2019). Although Syrians belong to the Middle Eastern ethnic group, limited data are available pertaining to the craniofacial characteristics of adolescents of Syrian nationality. Therefore, this compound cephalometric and tooth-size study was attempted to establish the dentofacial morphology, upper-airway dimensions, and tooth-size characteristics of Syrian adolescents with both skeletal and dental Class II-1 malocclusion. To our knowledge, this is the first comprehensive study on this topic.

In addition, 95% confidence intervals (CIs) for the means of cephalometric and tooth-size variables in the present study sample (Class II-1 malocclusion) (Table 1) were compared with 95% CIs for the means of normative cephalometric and tooth-size variables (Table S2) obtained from two previous studies (Nourallah et al., 2005; Al Sabbagh, 2014) and with 95% CIs for the means of the corresponding variables in previous Middle Eastern studies on Class II-1 malocclusion (Table S5).

### Skeletal components

In this study, the maxillary anteroposterior position was normal relative to the normative data, while the mandible was posteriorly positioned (CIs of A-NP, SNA, and Pog-NP in Table 1 vs. their corresponding values in Table S2); these findings were in agreement with those of several Middle Eastern studies (Demir, Uysal & Basciftci, 2005; Sayın & Türkkahraman, 2005; Isik et al., 2006; Mortazavi, Salehi & Ansari, 2009; Al Jundi & Riba, 2014) (CIs of A-NP, and Pog-NP in Table 1 vs. their corresponding values in Table S5). However, other Middle Eastern studies have reported different results (Al-Khateeb & Al-Khateeb, 2009; Ali, 2014); a possible reason for the divergence in findings could be, on the one hand, due to differences in the methods used for the determination of the maxillary and mandibular position (Pancherz, Zieber & Hoyer, 1997), while on the other hand, the involvement of different nationalities in the various studies may explain the range of results. Furthermore, previous Middle Eastern studies have suggested that the majority of Class II-1 patients may have abnormal development of the mandible, both in terms of size and in terms of position (Sayın & Türkkahraman, 2005; Mortazavi, Salehi & Ansari, 2009; Al Jundi & Riba, 2014). In this study, although a shorter absolute mandibular length was observed among Class II-1 subjects, the absolute maxillary length was also shorter when compared with the normative data, resulting in a normal maxillomandibular difference (CIs of Cond-A, Cond-Gn and Max-Mand in Table 1 vs. their corresponding values in Table S2). In the interpretation of such data, the difference between maxillary and mandibular lengths should also be considered because a geometric relationship exists between both measurements (McNamara, 1984). The normal maxillomandibular difference in this study confirms that mandibular length was proportional to the maxillary length. Therefore, the short mandibular and maxillary lengths do not represent a conclusive feature of Class II-1 subjects included in this study rather than a potential difference between this study and the cephalometric control study in estimating the point “condylion.” This point was considered by McNamara as “often difficult to find” and used as a measure of the lengths of both jaws; therefore, a slight difference in the estimation of condylion will simply affect the absolute lengths of both jaws but will not impact the maxillomandibular difference (McNamara, 1984).

**Figure 2:**
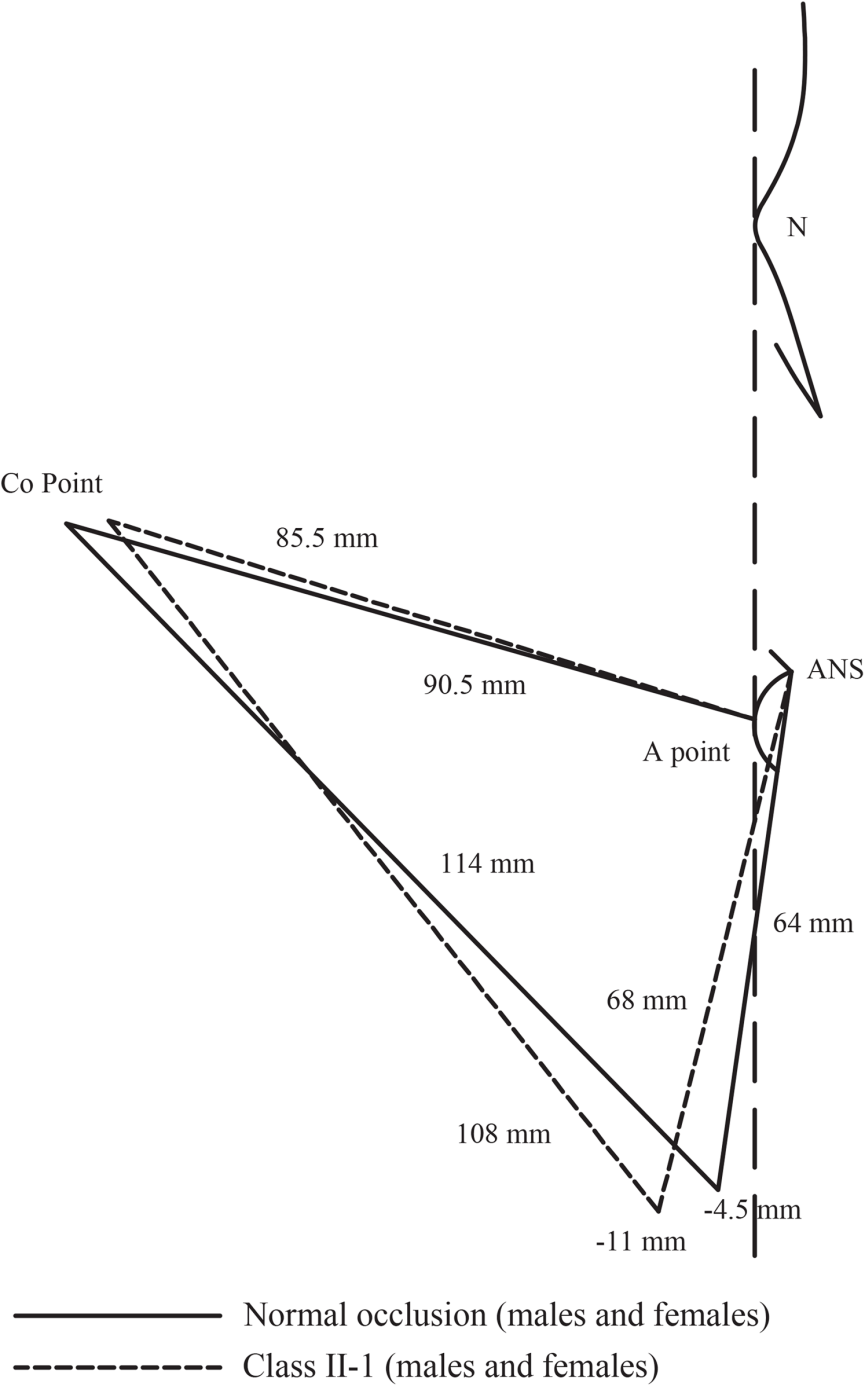
Comparison between Class II-1 subjects and normal occlusion subjects regarding the skeletal parameters.

A prominent feature of subjects with Class II-1 malocclusion in the present study was the hyperdivergent facial pattern. The retruded mandible appeared to be accompanied by an increased mandibular plane angle and opened facial axis in both genders and excessive lower anterior facial height that was clear enough in females (CIs of ANS-Me, MP-FH and Facial Axis in Table 1 vs. their corresponding values in Table S2). A review of the Middle Eastern literature suggests wide agreement with these results (Sayın & Türkkahraman, 2005; Isik et al., 2006; Al-Khateeb & Al-Khateeb, 2009; Mortazavi, Salehi & Ansari, 2009; Ali, 2014; Al Jundi & Riba, 2014) (CIs of MP-FH in Table 1 vs. their corresponding values in Table S5). Therefore, the posterior position of the mandible and, consequently, the inter-maxillary discrepancy of Class II-1 subjects in this study may be seen as a feature of the hyperdivergent facial pattern, as determined by the increased lower anterior facial height and backward rotation of the mandible (Fig. 2).

### Dentoalveolar components

Dentoalveolar aberrations were represented by protrusive mandibular incisors that were clear enough in females, whereas maxillary incisors were normally positioned in both genders when compared with the normative data (CIs of 1U-AP and 1L-APog in Table 1 vs. their corresponding values in Table S2). These findings contradicted those of certain Middle Eastern studies (Demir, Uysal & Basciftci, 2005; Ali, 2014). However, other Middle Eastern studies confirmed normally positioned maxillary incisors (Sayın & Türkkahraman, 2005) and more protrusive mandibular incisors existed in Class II-1 malocclusion (Al-Khateeb & Al-Khateeb, 2009; Mortazavi, Salehi & Ansari, 2009; Al Jundi & Riba, 2014) (CIs of 1U-AP and 1L-APog in Table 1 vs. their corresponding values in Table S5). The inconsistent findings between this study and other previous studies might be owing to the use of different reference lines for the determination of the incisors position or might be attributed to the variations in the nationality background of the studied samples.

In the present study, the protrusion of mandibular incisors in Class II-1 subjects (especially in females) might be attributed to the dentoalveolar compensatory mechanism in response to the underlying skeletal discrepancy attempting to maintain relatively normal relationships between the dental arches (Solow, 1980). Another explanation might be the relative tooth-size excess observed in the mandibular anterior segment (see below), since the protrusion of mandibular incisors could occur because of space limitations.

### Soft tissue components

The soft tissue parameters of Class II-1 subjects, when compared with the normative data, showed a more obtuse nasolabial angle (CIs of NLA in Table 1 vs. their corresponding values in Table S2). A previous Middle Eastern study found that Class II subjects had a greater value of the nasolabial angle than those with Class I; however, the difference was not significant (Gulsen et al., 2006). Conversely, other Middle Eastern studies suggested different results (Al-Saleem, 2013; Mohammed, Nissan & Taha, 2013) (CIs of NLA in Table 1 vs. their corresponding values in Table S5). A probable reason for this variability among outcomes might be the differences in the methods used to determine the nasolabial angle (Hwang, Kim & McNamara, 2000). Moreover, nationality differences may be a possible reason for such variability in soft tissue results.

Since there was no difference in the upper-lip angulation (CIs of UL-NP in Table 1 vs. their corresponding values in Table S2), our findings suggest that the angulation of the lower border of the nose may be a reason for the more obtuse nasolabial angle in Class II-1 subjects. In the present study, it was not possible to investigate the slope of the lower nasal border because there was no normative data available on this variable. Fitzgerald, Nanda & Currier (1992) established a reliable method of constructing the nasolabial angle to determine the interrelationship between the nose and the upper lip. Further investigations that consider using this method are needed to validate our results.

### Upper-airway dimensions

In the present study, upper-pharyngeal widths when compared with the normative data were smaller in Class II-1 subjects, and lower-pharyngeal widths showed smaller widths that were clear enough in males (CIs of UPh and LPh in Table 1 vs. their corresponding values in Table S2). These findings support the results of previous Middle Eastern studies (Ali, 2014; Elwareth Abd Elrazik Yousif, 2015; Gholinia, Habibi & Amrollahi Boyouki, 2019) (CIs of UPh and LPh in Table 1 vs. their corresponding values in Table S5). In contrast, some Middle Eastern studies denied that a relationship exists between Class II-1 malocclusion and airway dimensions (Abu Allhaija & Al-Khateeb, 2005; Uslu-Akcam, 2017). An explanation for the contrasting results might be an overlap between etiologic factors, including abnormal skeletal morphology and abnormal upper-airway soft tissue structures (Ferguson et al., 1995). In this study, the observed smaller pharyngeal widths might be attributable to the hyperdivergent facial pattern associated with Class II-1 malocclusion; this corroborates previous results in the Middle Eastern literature (Elwareth Abd Elrazik Yousif, 2015).

McNamara emphasized that an upper-pharyngeal width of 5 mm or less can be used as an indicator of possible airway obstruction, whereas lower pharyngeal measurements smaller than average values are not remarkable (McNamara, 1984). According to this indicator, there was no prevalence of pharyngeal obstructions in the subjects of this study. However, a more accurate diagnosis can be made only by an otorhinolaryngologist (McNamara, 1984).

### Tooth-size characteristics

Former Middle Eastern studies have indicated that tooth-size disharmonies exist among different groups of malocclusion (Fattahi, Pakshir & Hedayati, 2006; Mollabashi et al., 2019). In accordance with our results on tooth-size ratios (CIs of anterior ratio and overall ratio in Table 1 vs. their corresponding values in Table S2), some Middle Eastern studies did not find differences in overall ratios between Class II-1 subjects and the normative data (Asiry & Hashim, 2012), whereas other Middle Eastern studies described an overall ratio for Class II-1 subjects that was smaller (Mollabashi et al., 2019; Shamaa, 2019) or larger (Uysal et al., 2005) compared to that of subjects with normal occlusion. Similarly, variability can be found in the Middle Eastern literature regarding the anterior ratio, whereas a smaller ratio (Fattahi, Pakshir & Hedayati, 2006) or no differences were detected (Uysal et al., 2005; Asiry & Hashim, 2012) (CIs of anterior ratio and overall ratio in Table 1 vs. their corresponding values in Table S5). Such divergence in findings may be explained by differences in the nationality background of the samples.

Several studies confirmed that a tooth-size disharmony greater than two SDs, as compared with Bolton’s norms, could create clinical difficulties, particularly in the finishing phase of treatment (Crosby & Alexander, 1989; Wędrychowska-Szulc, Janiszewska-Olszowska & Stepień, 2010). In contrast, one study suggested that Bolton’s SDs may not be a valuable index to use to determine the clinical significance of tooth-size disharmony because of their relatively modest values (Othman & Harradine, 2006). Therefore, the frequencies of tooth-size discrepancy outside two SDs from Bolton’s norms as well as the frequencies of tooth-size discrepancy outside two SDs from Syrian population norms were calculated in this study. The percentage of patients with anterior ratios greater than two SDs from Bolton’s norm was 39.5%, while the percentage of patients who had anterior ratios greater than two SDs from Syrian population norm was 25.6%, representing a relative tooth-size excess in the mandibular anterior segment that was great enough to warrant clinical concern. These relatively high percentages of patients with an anterior ratio exceeding two SDs may be explained by the association of a higher percentage of tooth-size disharmonies and sufficiently remarkable malocclusions such as Class II-1 malocclusion. These disharmonies are particularly evident in the anterior segment, since the anterior teeth, especially the incisors, have the greatest incidence of tooth-size variations (Fattahi, Pakshir & Hedayati, 2006).

### Limitations

As far as limitations of the current study were concerned, consideration must be given to the sample size. Although the sample size estimation showed sufficient sample size for each gender group, the sample size was small; therefore, the results should be interpreted with caution and further studies with larger sample size are warranted. Such studies should also include adolescents from several orthodontic centers, since the data in the present study were recruited from only one private orthodontic practice in Syria. Additionally, there was no concurrent control group in this study. The inclusion of a matched control group in this investigation would have been desirable for a better comparison, although, in the case of normative cephalometric data, requiring the exposure of patients with well-balanced dentofacial relationships to *X*-ray radiation constitutes an ethical issue. As an alternative, already established cephalometric and tooth-size data were used as sources of normative measurements (Nourallah et al., 2005; Al Sabbagh, 2014). Although age could not be a confounding factor in tooth-size analysis, a wider age range was used in the normative cephalometric study than that in this study which could be a confounding factor in the cephalometric analysis. Because no other cephalometric data have been published on Syrian adolescent norms; these published data, therefore, were used as sources of normative measurements.

## Conclusions

- In this study, cephalometric results showed that a hyperdivergent facial pattern was the main cause of the inter-maxillary discrepancy in Syrian adolescents with Class II-1 malocclusion, while the observed small pharyngeal widths were not clinically significant.
- Tooth-size results revealed that 39.5% of samples had anterior ratios exceeding two SDs of Bolton’s norm and 25.6% of samples had anterior ratios exceeding two SDs of Syrian population norm, which may be considered as clinically relevant.
- Determining the craniofacial characteristics of Class II-1 malocclusion in the young Syrian population would help orthodontists to establish an effective protocol for long-term stable treatment of Syrian orthodontic patients.

## Supplemental Information

10.7717/peerj.9545/supp-1 Supplemental Information 1 Distribution of Class II-1 malocclusion in different populations. *Mean age. **Patients with subdivision Class II-1 malocclusion were included. ***Calculated percentage based on the study results. †Male sample.

10.7717/peerj.9545/supp-2 Supplemental Information 2 Normative cephalometric measurements and tooth-size ratios (Nourallah et al., 2005; Al Sabbagh, 2014). CIs, confidence intervals, S.D, standard deviation.

10.7717/peerj.9545/supp-3 Supplemental Information 3 Age and gender distributions in the present study sample and normative studies’ samples. ND indicates not declared.

10.7717/peerj.9545/supp-4 Supplemental Information 4 Definitions of the cephalometric measurements used in the present study.

10.7717/peerj.9545/supp-5 Supplemental Information 5 Corresponding cephalometric measurements and tooth-size ratios in previous Middle Eastern studies on Class II-1 malocclusion. CIs, confidence intervals, M, males, F, females*Class II malocclusion

10.7717/peerj.9545/supp-6 Supplemental Information 6 Cephalometric measurements and tooth-size ratios.

10.7717/peerj.9545/supp-7 Supplemental Information 7 Tooth-size measurements.

## Additional Information and Declarations

### Data Availability

The following information was supplied regarding data availability: The raw measurements are available in the Supplemental Files.
