# Supplementary material for: Craniofacial characteristics of Syrian adolescents with Class II division 1 malocclusion: a retrospective study
Source: PeerJ. 2020 Jul 15;8:e9545. doi: 10.7717/peerj.9545 (PMC7368432; doi:10.7717/peerj.9545)
Supplement: Supplemental Information 1 — *Mean age. **Patients with subdivision Class II-1 malocclusion were included. ***Calculated percentage based on the study results. †Male sample. [file peerj-08-9545-s001.docx]

**Table S1:** Distribution of Class II-1 malocclusion in different populations.

| Series | Population | Size (n) | Age (y) | Class II-1 (%) |
| --- | --- | --- | --- | --- |
| (Lew, Foong & Loh, 1993) | Chinese | 1050 | 12-14 | 18.8 |
| (Foster & Walpole Day, 1974) | British | 1000 | 11-12 | 27.2 |
| (Onyeaso, 2004) | Nigerian | 636 | 12-17 | 12.3 |
| (Borzabadi-Farahani, Borzabadi-Farahani & Eslamipour, 2009) | Iranian | 502 | 11-14 | 24.1 |
| (Thilander et al., 2001) | Colombian | 4724 | 5-17 | 14.9 |
| (Bilgic, Gelgor & Celebi, 2015) | Turkish | 2329 | 12-16 | 40.0 |
| (Perillo et al., 2009) | Italian | 703** | 12.2* | 33.7 |
| (Albakri, Ingle & Assery, 2018) | Saudi | 500† | 12-15 | 17.4 |
| (Tausche, Luck & Harzer, 2004) | German | 1975 | 6-8 | 31.4 |
| (Silva & Kang, 2001) | Latin American | 507 | 12-18 | 20.3 |
| (Saleh, 1999) | Lebanese | 851 | 9-15 | 16.9 |
| (Massler & Frankel, 1951) | American | 2728 | 14-18 | 16.68 |
| (Gábris, Márton & Madléna, 2006) | Hungarian | 483 | 16-18 | 25.9 |
| (Shyagali et al., 2019) | Indian | 171 | 12-16 | 21.05 |
| (Bugaighis & Karanth, 2013) | Libyan | 343 | 12-17 | 21.9 |
| (Nadim, Aslam & Rizwan, 2014) | Pakistani | 718 | 12-15 | 17.8 |
| (El-Mangoury & Mostafa, 1990) | Egyptian | 501 | 18-24 | 16.17 |
| (Thilander & Myrberg, 1973) | Swedish | 6398 | 7-13 | 13.23*** |
| (Singh & Sharma, 2014) | Nepalese | 2010 | 12-15 | 29.35 |
| (de Souza et al., 2016) | Brazilian | 1014 | 7-11 | 21 |
| (Alatrach, Saleh & Osman, 2014) | Syrian | 200 | 8-13 | 16 |

*Mean age.

**Patients with subdivision Class II-1 malocclusion were included.

***Calculated percentage based on the study results.

†Male sample.
