# Supplementary material for: Craniofacial characteristics of Syrian adolescents with Class II division 1 malocclusion: a retrospective study
Source: PeerJ. 2020 Jul 15;8:e9545. doi: 10.7717/peerj.9545 (PMC7368432; doi:10.7717/peerj.9545)
Supplement: Supplemental Information 2 — CIs, confidence intervals, S.D, standard deviation. [file peerj-08-9545-s002.docx]

**Table S2:** Normative cephalometric measurements and tooth-size ratios (Nourallah et al., 2005; Al Sabbagh, 2014).

| **Cephalometric measurements** | **Syrian Males with Normal Occlusion (n = 50)** | | **Syrian Females with Normal Occlusion (n = 50)** | | **Syrian adolescents with Normal Occlusion (n = 100)** | |
| --- | --- | --- | --- | --- | --- | --- |
| **Variables** | Mean (±S.D) | 95% CIs for mean  Lower, Upper | Mean (±S.D) | 95% CIs for mean  Lower, Upper | Mean (±S.D) | 95% CIs for mean  Lower, Upper |
| Skeletal measurements |  | | | | | |
| Sagittal values |  | | | | | |
| A-NP (mm) | -1.14 (±1.28) | **-1.51, -0.77** | -0.04 (±1.34) | **-0.42, 0.34** | -0.58 (±0.96) | **-0.77, -0.39** |
| SNA (°) | 80.26 (±2.72) | **79.48, 81.04** | 80.87 (±2.49) | **80.16, 81.58** | 80.58 (±1.63) | **80.25, 80.91** |
| Pog-NP (mm) | -4.72 (±5.77) | **-6.37, -3.07** | -4.12 (±5.62) | **-5.73, -2.51** | -4.41 (±2.09) | **-4.83, -3.99** |
| Cond-A (mm) | 90.76 (±3.61) | **89.73, 91.79** | 90.34 (±3.72) | **89.28, 91.40** | 90.61 (±2.14) | **90.18, 91.04** |
| Cond-Gn (mm) | 114.77 (±4.65) | **113.44, 116.10** | 113.16 (±4.88) | **111.77, 114.55** | 113.98 (±1.98) | **113.58, 114.38** |
| Max-Mand (mm) | 24.01 (±3.77) | **22.93, 25.09** | 22.72 (±3.18) | **21.81, 23.63** | 23.38 (±2.16) | **22.95, 23.81** |
| Vertical values |  | | | | | |
| ANS-Me (mm) | 65.04 (±4.82) | **63.66, 66.42** | 62.57 (±4.80) | **61.20, 63.94** | 63.82 (±2.50) | **63.32, 64.32** |
| MP-FH (°) | 23.01 (±4.48) | **21.73, 24.29** | 22.19 (±5.11) | **20.73, 23.65** | 22.61 (±2.16) | **22.18, 23.04** |
| Facial Axis (°) | 1.17 (±3.34) | **0.22, 2.12** | 0.46 (±4.09) | **-0.71, 1.63** | 0.83 (±2.26) | **0.38, 1.28** |
| Dental measurements |  | | | | | |
| 1U-AP (mm) | 5.82 (±2.36) | **97.55, 99.17** | 5.26 (±2.52) | **4.54, 5.98** | 5.55 (±1.20) | **5.31, 5.79** |
| 1L-APog (mm) | 3.13 (±2.28) | **2.48, 3.78** | 2.53 (±2.36) | **1.86, 3.20** | 2.84 (±1.31) | **2.58, 3.10** |
| Soft tissue measurements |  | | | | | |
| NLA (°) | 98.36 (±2.85) | **97.55, 99.17** | 97.14 (±2.21) | **96.51, 97.77** | 97.76 (±1.69) | **97.42, 98.10** |
| UL-NP (°) | 9.02 (±3.28) | **8.08, 9.96** | 13.53 (±3.85) | **12.43, 14.63** | 11.29 (±0.84) | **11.12, 11.46** |
| Airway measurements |  | | | | | |
| UPh (mm) | 17.65 (±3.11) | **16.76, 18.54** | 16.96 (±2.87) | **16.14, 17.78** | 17.32 (±1.36) | **17.05, 17.59** |
| LPh (mm) | 13.44 (±2.56) | **12.71, 14.17** | 13.01 (±2.25) | **12.37, 13.65** | 13.24 (±1.21) | **13.00, 13.48** |
|  | | | | | | |
| **Tooth-size ratios** | **Syrian Males with Normal Occlusion (n = 35)** | | **Syrian Females with Normal Occlusion (n = 20)** | | **Syrian adolescents with Normal Occlusion (n = 55)** | |
| **Variables** | Mean (±S.D) | 95% CIs for means  Lower, Upper | Mean (±S.D) | 95% CIs for means  Lower, Upper | Mean (±S.D) | 95% CIs for means  Lower, Upper |
| Anterior ratio (%) | 78.90 (±2.29) | **78.11, 79.69** | 79.20 (±2.02) | **78.25, 80.15** | 78.99 (±2.18) | **78.40, 79.58** |
| Overall ratio (%) | 92.40 (±2.06) | **91.69, 93.11** | 91.90 (±2.08) | **90.93, 92.87** | 92.26 (±2.06) | **91.70, 92.82** |

**CIs=confidence intervals, S.D=standard deviation.**
