# Supplementary material for: Craniofacial characteristics of Syrian adolescents with Class II division 1 malocclusion: a retrospective study
Source: PeerJ. 2020 Jul 15;8:e9545. doi: 10.7717/peerj.9545 (PMC7368432; doi:10.7717/peerj.9545)
Supplement: Supplemental Information 3 — ND indicates not declared. [file peerj-08-9545-s003.docx]

**Table S3:** Age and gender distributions in the present study sample and normative studies' samples.

|  | Sample size (n) | Mean age ± S.D (y) | Age range (y) |
| --- | --- | --- | --- |
| Class II-1 sample |  |  |  |
| Female | 24 | 14.6 ± 1.4 | 11.9 – 17.1 |
| Male | 19 | 14.1 ± 1.7 | 11.7 - 17.3 |
| Total | 43 | 14.3 ± 1.5 | 11.7 - 17.3 |
| Normative tooth-size sample |  |  |  |
| Female | 20 | ND | ND |
| Male | 35 | ND | ND |
| Total | 55 | ND | 11 - 22 |
| Normative cephalometric sample |  |  |  |
| Female | 50 | 16.4 ± 2.1 | ND |
| Male | 50 | 16.7 ± 1.7 | ND |
| Total | 100 | 16.6 ± 1.9 | 13.5 - 20 |

ND indicates not declared.
