# Supplementary material for: Craniofacial characteristics of Syrian adolescents with Class II division 1 malocclusion: a retrospective study
Source: PeerJ. 2020 Jul 15;8:e9545. doi: 10.7717/peerj.9545 (PMC7368432; doi:10.7717/peerj.9545)
Supplement: Supplemental Information 4 [file peerj-08-9545-s004.docx]

**Table S4:** Definitions of the cephalometric measurements used in the present study.

| Skeletal measurements |  |
| --- | --- |
| Sagittal values |  |
| 1. A-NP | Distance from point A to Nasion perpendicular. |
| 1. SNA | Angle formed by connecting the Sella-Nasion plane to point A. |
| 1. Pog-NP | Distance from point Pog to Nasion perpendicular. |
| 1. Cond-A | Effective Length of Maxilla: Distance from point Condylion to point A. |
| 1. Cond-Gn | Effective Length of Mandible: Distance from point Condylion to point anatomic Gn. |
| 1. Max-Mand | Maxillomandibular difference: Difference between effective maxillary and mandibular lengths. |
| Vertical values |  |
| 1. ANS-Me | Distance from point ANS (anterior nasal spine) to point Me. |
| 1. MP-FH | Mandibular Plane Angle: Angle between the mandibular plane (Me-Go) and the anatomic Frankfurt plane (P-Or). |
| 1. Facial Axis | Angle formed by the reference lines N-Ba (cranial base) and PTM- constructed Gn (facial axis). The value measured is Ba-PTM-cGn angle - 90° |
| Dental measurements |  |
| 1. 1U-AP | Distance from the most anterior surface of the upper incisor to a vertical line to Frankfurt plane through point A. |
| 1. 1L-APog | Distance from the most anterior surface of the lower incisor to the line A-Pog. |
| Soft tissues measurements |  |
| 1. NLA | Naso-labial angle: Angle formed by the tangent from Subnasale (Sn) to the inferior aspect of the nose and the tangent to the upper lip. |
| 1. UL-NP | Angle formed by the tangent drawn to the upper lip and the Nasion perpendicular. |
| Airway measurements |  |
| 1. UPh | Upper pharyngeal width: The closest distance from the anterior half of the posterior outline of the soft palate to the posterior pharyngeal wall. |
| 1. LPh | Lower pharyngeal width: The closest distance from the intersection of the posterior border of the tongue and the inferior border of the mandible to the posterior pharyngeal wall. |
