# Supplementary material for: Craniofacial characteristics of Syrian adolescents with Class II division 1 malocclusion: a retrospective study
Source: PeerJ. 2020 Jul 15;8:e9545. doi: 10.7717/peerj.9545 (PMC7368432; doi:10.7717/peerj.9545)
Supplement: Supplemental Information 5 — CIs, confidence intervals, M, males, F, females*Class II malocclusion [file peerj-08-9545-s005.docx]

**Table S5:** Corresponding cephalometric measurements and tooth-size ratios in previous Middle Eastern studies on Class II-1 malocclusion.

| **Cephalometric measurements** | | **Means (95% CIs for means)** | | | | | | | |
| --- | --- | --- | --- | --- | --- | --- | --- | --- | --- |
| **Nationality & Series / Variables** | | **A-NP (mm)** | **Pog-NP (mm)** | **MP-FH (°)** | **1U-AP (mm)** | **1L-APog (mm)** | **NLA (°)** | **UPh (mm)** | **LPh (mm)** |
| **Turkish** (Sayın & Türkkahraman, 2005) | F | 0.9  (-0.2, 2.0) | -10.6  (-13.2, -8.0) | 28.3  (25.9, 30.7) | 5.9  (5.1, 6.7) | 2.2  (1.3, 3.1) | - | - | - |
| **Iraqi**  (Ali, 2014) | M | 2.2  (2.0, 2.5) | -7.4  (-8.1, -6.6) | 34.0  (33.0, 34.9) | 8.5  (7.9, 9.0) | 2.8  (2.4, 3.2) | - | 13.4  (12.6, 14.2) | 9.8  (9.2, 10.4) |
|  | F | 2.7  (2.5, 2.9) | -8.7  (-9.5, -7.8) | 34.6  (32.9, 36.3) | 7.6  (6.9, 8.4) | 2.8  (2.5, 3.1) | - | 13.2  (12.5, 13.9) | 8.4  (7.8, 9.0) |
| **Egyptian**  (ElAbbasy, 2019) | F | 0.7  (-1.3, 2.7) | -5.5  (-8.9, -2.1) | - | - | - | - | - | - |
| **Saudi**  (Al Jundi & Riba, 2014) | M | - | - | - | - | 3.4  (2.9, 3.8) | - | - | - |
|  | F | - | - | - | - | 3.5  (3.1, 3.8) | - | - | - |
| **Egyptian**  (Fouda, Hafez & Al-Awdi, 2017) | M  +  F | - | - | **-** | **-** | - | 123.8  (118.9, 128.6) | - | - |
| **Iraqi***  (Mohammed, Nissan & Taha, 2013) | M | - | - | **-** | **-** | - | 105.1  (104.2, 105.9) | - | - |
|  | F | - | - | **-** | **-** | - | 110.6  (109.6, 111.6) | - | - |
| **Turkish***  (Gulsen et al., 2006) | M  +  F | - | - | **-** | **-** | - | 108.5  (105.3, 111.7) | - | - |
| **Iranian***  (Gholinia, Habibi & Amrollahi Boyouki, 2019) | M  +  F | - | - | **-** | **-** | - | - | - | 9.6  (8.4, 10.9) |
|  | |  | | | | | | | |
| **Tooth-size ratios** | | **Means (95% CIs for means)** | | | | | | | |
| **Nationality & Series / Variables** | | **Anterior ratio** | | | | **Overall ratio** | | | |
| **Egyptian***  (Ali, El-Shorbagy & Elliathy, 2016) | M | 78.7  (77.7, 79.7) | | | | 92.5  (91.9, 93.1) | | | |
|  | F | 78.4  (77.6, 79.1) | | | | 91.9  (91.2, 92.6) | | | |
| **Saudi**  (Asiry & Hashim, 2012) | M | 77.9  (77.1, 78.7) | | | | 92.1  (91.3, 92.8) | | | |
|  | F | 77.4  (76.2, 78.6) | | | | 91.8  (91.0, 92.6) | | | |
| **Iranian** (Mollabashi et al., 2019) | M  +  F | 78.5  (77.8, 79.3) | | | | 91.6  (91.0, 92.2) | | | |

**CIs=confidence intervals, M=males, F=females**

***Class II malocclusion**
